# Cerebral cortical encephalitis in adults with myelin oligodendrocyte glycoprotein antibody‐associated disease: A national case series

**DOI:** 10.1111/ene.16550

**Published:** 2024-11-19

**Authors:** Samuel Pace, Silvia Messina, Bo Chen, Radu Tanasescu, Athanasios Papathanasiou, Cris S. Constantinescu, Maria I. Leite, Mark D. Willis, Janet A. Johnston, Jacqueline Palace, Ruth Dobson

**Affiliations:** ^1^ Department of Neurology Royal London Hospital, Barts Health NHS Trust London UK; ^2^ Nuffield Department of Clinical Neurosciences Oxford University Hospitals Oxford UK; ^3^ Neurology Department, Wexham Park Hospital Frimley Foundation Health Trust Slough UK; ^4^ Department of Neurology, Tongji Hospital of Tongji Medical College Huazhong University of Science and Technology Wuhan China; ^5^ Mental Health and Clinical Neuroscience Academic Unit University of Nottingham School of Medicine Nottingham UK; ^6^ Department of Neurology Nottingham University Hospitals NHS Trust Nottingham UK; ^7^ Cooper Neurological Institute Cooper Medical School of Rowan University Camden New Jersey USA; ^8^ Helen Durham Centre for Neuroinflammatory Disease, Department of Neurology University Hospital of Wales Cardiff UK; ^9^ Department of Neurology University Hospital of Wales Cardiff UK; ^10^ Centre for Preventive Neurology Wolfson Institute of Population Health, Queen Mary University London London UK

**Keywords:** cerebral cortical encephalitis, MOG antibody disease, MRI findings, neuromyelitis optica spectrum disorder

## Abstract

**Background and purpose:**

Myelin oligodendrocyte glycoprotein (MOG) antibody‐associated disease (MOGAD) is a relatively recently described disease, most commonly presenting with optic neuritis and longitudinally extensive transverse myelitis. Cerebral cortical encephalitis is a rare manifestation of MOGAD.

**Methods:**

We identified patients presenting with cerebral cortical encephalitis with positive MOG antibodies in serum across a large specialized service. Demographic and clinical information were collected. We describe clinical and laboratory characteristics, treatment response, and subsequent relapse risk in adults presenting with this phenotype.

**Results:**

We identified eight patients meeting clinical criteria for cerebral cortical encephalitis with MOG antibodies. All had seizures; four had focal onset seizures with or without secondary generalization. Two patients exhibited encephalopathy, and six demonstrated focal neurological deficits at presentation. All had fluid‐attenuated inversion recovery hyperintensities. Five of eight displayed cerebral swelling, and two of eight displayed leptomeningeal enhancement. Where cerebrospinal fluid (CSF) results were available, five of seven had CSF pleocytosis, protein was raised in two of seven, and one patient had oligoclonal bands unique to CSF. Median time to seizure control was 1.25 months, and all clinical features and magnetic resonance imaging abnormalities resolved. Four of eight patients (50%) had a clinical relapse, with a median time to relapse of 6.4 months.

**Conclusions:**

Cerebral cortical encephalitis appears to share similar CSF findings, steroid responsiveness, and risk of relapse with other clinical manifestations of MOGAD. This informs treatment decisions and patient counselling.

## INTRODUCTION

Myelin oligodendrocyte protein (MOG), an oligodendrocyte membrane protein [1], is a target for IgG‐mediated inflammation. It was first identified in patients presenting with features of neuromyelitis optica spectrum disorder (NMOSD) who were aquaporin‐4 antibody seronegative [2]. Over time, the neurological syndromes, disease course, treatment responses, and prognosis associated with positive MOG antibodies have been increasingly described and refined. This has led to the definition of a distinct disease entity, MOG antibody‐associated disease (MOGAD) [3], with specific histopathology now described [4].

Patients with MOGAD may only experience a single demyelinating episode during their lifetime. Alternatively, a relapsing phenotype may emerge [5]. MOGAD is associated with a spectrum of clinical phenotypes; patients typically present with acute disseminated encephalomyelitis (ADEM), optic neuritis (ON), or transverse myelitis (TM) [6, 7, 8], and less commonly with encephalitis or with features of brainstem or cerebellar involvement [9, 10].

Cerebral cortical encephalitis is a rare manifestation of MOGAD, first reported in 2017 [9]. This condition typically presents with headache, seizures, fever, and focal cortical symptoms. It has also been termed “fluid‐attenuated inversion recovery (FLAIR) hyperintense lesions in anti‐MOG‐associated encephalitis with seizures” (FLAMES) when seizures are a clinical feature [11]. Magnetic resonance imaging (MRI) reveals T2 FLAIR hyperintensity and cortical swelling sometimes associated with leptomeningeal enhancement. Cerebrospinal fluid (CSF) analysis often identifies a pleocytosis. Both the clinical features and imaging findings have been reported to improve with corticosteroid treatment [9, 11]. Cerebral cortical encephalitis has been reported in both children and adults, with some evidence suggesting a higher prevalence in the paediatric cohort [12]. Presentations within the adult cohort with MOGAD are rare, and current knowledge has originated from individual case reports with limited follow‐up.

In this case series, we use a large database from a highly specialized service alongside active case ascertainment across neurology centres to understand the treatment response and subsequent relapse risk in adults presenting with cerebral cortical encephalitis. Through this, we aim to inform counselling of patients and future clinical treatment strategies.

## METHODS

### Study design and participants

This cohort study included retrospectively identified patients who were MOG antibody positive on a live cell‐based assay, and who presented with a clinical and radiological syndrome meeting criteria for a diagnosis of cerebral cortical encephalitis at the time of MOG antibody positivity, with onset in adulthood [9]. A national clinical database, the Oxford NMOSD database, was used to identify adult patients with MOGAD who had a recorded “brain attack” (ADEM) presentation, and/or recorded seizures in the context of presumed or confirmed relapse. Additional patients were identified via direct outreach to sites both that contribute and that do not contribute patients to the Oxford NMOSD database.

Patients from the Oxford NMOSD database signed written informed consent according to Oxford Research Ethics (Research Ethics Committee C Ref: 10/H0606/5); where patients had not provided written informed consent for the central database and biobanking, consent was obtained and recorded in the clinical notes for inclusion in this case series.

### Clinical data collection

Demographic and clinical information was collected for each patient. Data collected included age at onset, sex, ethnicity, clinical features, preceding and subsequent clinical manifestations of MOGAD, comorbidities, MRI findings, CSF analysis, immunotherapy and steroid exposure, serum MOG IgG status, N‐methyl‐D‐aspartate (NMDA) receptor antibody status, antiseizure medications, and clinical and radiological response to treatment.

## RESULTS

### Demographic and clinical features

We identified eight adult patients who met criteria for cerebral cortical encephalitis with MOG antibodies [9]. Median age at onset was 32.5 (interquartile range [IQR] = 24–38.5) years, with a male‐to‐female ratio of 6:2. All patients presented in adulthood, with the youngest age at presentation being 21 years. The prevalence of cerebral cortical encephalitis in patients with MOGAD from the Oxford NMOSD database was 2.78% (five in 180 patients). Four of eight were of Asian ethnicity (South and Southeast Asian), one was Black Caribbean–British, and three were of White ethnicity. Three patients had preceding headache recorded in the clinical notes, and one had a documented preceding fever.

Seven of eight (88%) patients presented with generalized tonic–clonic seizures, with four (50%) having focal onset seizures with or without secondary generalization. Two (25%) patients exhibited encephalopathy, and six (75%) demonstrated focal neurological deficits at presentation; two (25%) were dysarthric, two (25%) exhibited facial palsy, three (38%) were ataxic, one (13%) had diplopia, and one (12%) had ptosis. Only one patient had preceding ON, and no patients had preceding TM.

An additional patient, for whom limited clinical information was available, was also identified. This patient presented with focal and generalized tonic–clonic seizures, and historic MRI was reported to be abnormal, although no formal report or imaging was available. Control of her seizures was achieved with intravenous methylprednisolone and phenytoin. She later relapsed with ON. Data from this patient were not included in further reporting.

### Paraclinical features

**FIGURE 1:**
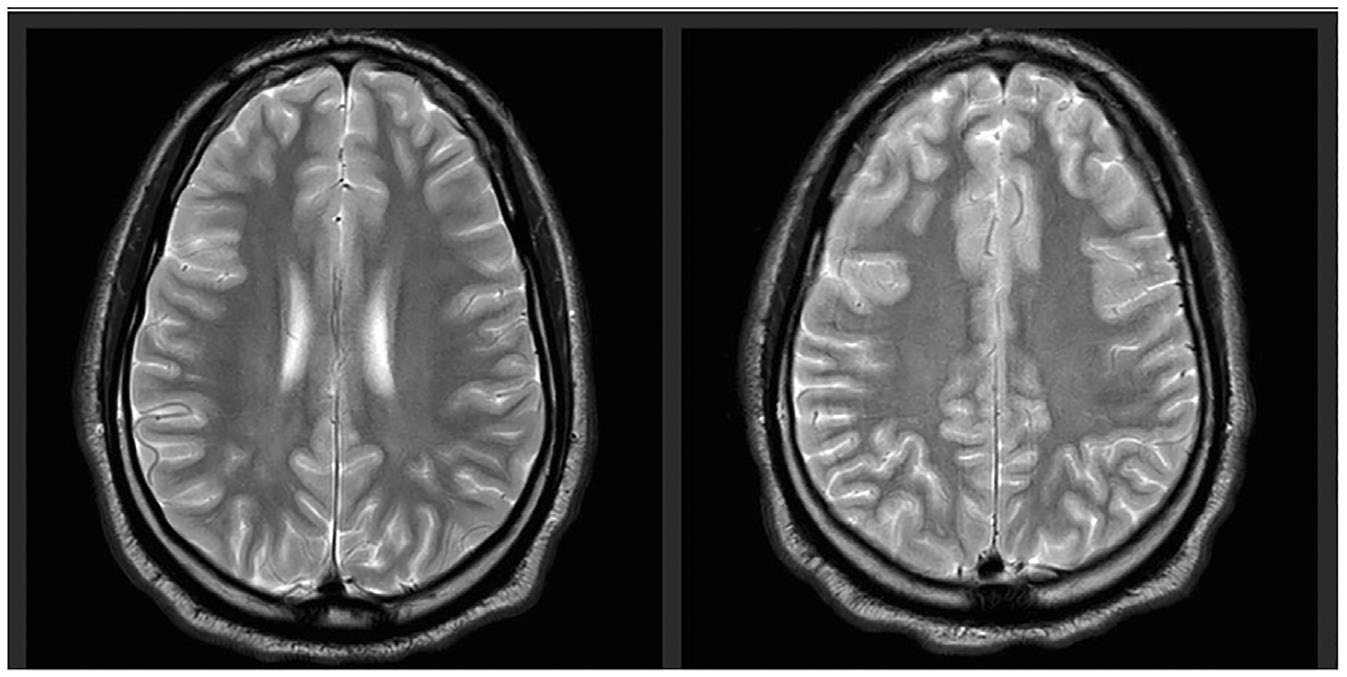
Axial T2‐weighted magnetic resonance images demonstrating cortical swelling with hyperintense T2 signal affecting frontal and parietal lobes, changes typical of those seen in the context of cerebral cortical encephalitis.

Formal MRI reports were available for all patients. All had FLAIR hyperintensities (see Figure 1). Five of eight (63%) displayed cerebral swelling, and two of eight (25%) demonstrated leptomeningeal enhancement. Six of eight (75%) had abnormalities restricted to one hemisphere; two of eight (25%) had abnormalities bilaterally. No patients had concomitant white matter lesions on their MRI scans.

CSF was examined in seven of eight patients (88%; see Table 1). CSF pleocytosis was found in five of seven (71%), with median cell count of 10.7 cells/mm^3^ (range = 0–198). CSF protein was raised in two of seven (29%), with median protein concentration of 447 mg/L (range = 308–816). Only one patient had oligoclonal bands (OCBs) unique to the CSF; these were of type II pattern. Opening pressure was measured and recorded in one patient only and was raised. NMDA receptor antibody status was negative in five of five patients tested.

**TABLE 1:** CSF findings in patients with cerebral cortical encephalitis.

CSF analysis	Value
White cell count, cells/mm^3^	Mean = 40
	Median = 10.7
	Range = 0–198
Pleocytosis, *n*	5/7
Protein, mg/L	Mean = 479
	Median = 447
	Range = 308–816
Raised protein, *n*	2/7
Oligoclonal bands present on isoelectric focusing with immunofixation, *n*	1/7

Abbreviation: CSF, cerebrospinal fluid.

Interictal electroencephalographic (EEG) testing was performed on five patients; two (40%) were abnormal, both of whom showed widespread cerebral dysfunction.

### Treatments and clinical progress

Five of eight (63%) patients were treated acutely with high‐dose methylprednisolone, four intravenously and one orally. Five (63%) were given a prednisolone taper, and three (38%) received additional immunosuppression with either mycophenolate (*n* = 2) or azathioprine (*n* = 1). Three patients did not receive any steroids acutely, and two did not receive a taper.

Clinical outcomes are summarized in Table 2. All patients received antiseizure medications acutely. Seven (88%) were given levetiracetam monotherapy, and one (13%) received carbamazepine and clobazam in addition to levetiracetam. All patients had symptom resolution, including control of seizures. Median time to seizure control was 1.25 months (IQR = 0.25–1.63, range = 0–7). One patient who did not receive any steroids took 7 months to achieve seizure control. Two of eight (25%) had their antiseizure medication successfully weaned or stopped.

**TABLE 2:** Clinical outcomes following presentation with cerebral cortical encephalitis.

Clinical progress	*n*
Symptom resolution	8/8
Antiepileptic dose reduction	1/8
Antiepileptic cessation	1/8
Improved MRI changes on repeated imaging	6/6
Repeat MOG serology positive	4/7
Clinical relapse (with relapse phenotype)	4/8 Cerebral cortical encephalitis (*n* = 3) Optic neuritis (*n* = 2) Transverse myelitis (*n* = 1) Area postrema syndrome (*n* = 1)

Abbreviations: MOG, myelin oligodendrocyte protein; MRI, magnetic resonance imaging.

Follow‐up MRI was available for six patients. All showed improvement of MRI changes on repeated imaging after treatment. Repeat MOG serology was positive in four of the seven (57%) patients in whom it was tested; as all tests were not performed using the same standardized assay, titres are not reported.

Four of eight patients (50%) had a clinical relapse, with a median time to relapse of 6.4 months. Three patients had a relapse consistent with recurrence of cerebral cortical encephalitis, one of whom also developed ON, TM, and area postrema syndrome, and one other patient developed new ON. The patients who relapsed with a phenotype consistent with cerebral cortical encephalitis presented with focal seizures, one with secondary generalization, with FLAIR hyperintensities on MRI. One had leptomeningeal enhancement, and one had diffusion restriction. One patient was treated with intravenous methylprednisolone and plasma exchange, whereas the other two received oral methylprednisolone followed by a steroid taper. Two had their antiseizure medication dosage increased, and one received augmentation with a second agent. EEG was performed in two patients, which revealed epileptogenic activity in one, corresponding to their FLAIR hyperintensity. Time to seizure control for those with a cerebral cortical encephalitis relapse was 1 day in one patient and 1 week in the other; in both, this was more rapid than at initial presentation. The clinical course of the third patient was somewhat different, characterized by recurrent seizure relapses. At hospital discharge, two patients were receiving steroid‐sparing agents (azathioprine and mycophenolate mofetil), and the third received a course of prednisolone. Two had normal follow‐up MRI scans, whereas one showed persistent cerebral inflammation on serial scans. The patient who experienced TM, ON, and cerebral cortical encephalitis achieved good control with the addition of rituximab and has not relapsed in the 6 years since initiation.

MOG antibodies were retested in all four patients who relapsed. One patient who relapsed remained anti‐MOG seropositive, and one became and stayed anti‐MOG low positive, whereas two were seronegative.

## DISCUSSION

Cerebral cortical encephalitis has emerged as a distinct clinical manifestation of MOGAD, with key features described in this case series. As would be expected, seizures were highly prevalent; all patients experienced generalized tonic–clonic seizures. It is notable that a majority also experienced focal seizures. Encephalopathy and focal neurological deficits were also commonly reported in this cohort. Imaging revealed evidence of cerebral swelling in the majority, as has been highlighted in previous case reports of cerebral cortical encephalitis [9, 11]. Although CSF results varied individually, the very low frequency of OCBs is notable, along with pleocytosis in more than half.

Recent work has highlighted the range of clinical manifestations of MOGAD, with the most common clinical features in adults being ON (seen in 55%–60% of adults with MOGAD) and TM (seen in 25%–30% of adults) [3]. ADEM or brain lesions are only seen in approximately 10% of adults, in contrast to up to 50% of children; however, in the majority, brain lesions do not meet the criteria for cerebral cortical encephalitis/FLAMES, instead tending to be large, bilateral, and ill‐defined, often involving deep grey matter and pons [3]. Similar to paraclinical findings in other clinical manifestations of MOGAD, oligoclonal bands were rarely seen in our cohort, and CSF pleocytosis was seen in some, but not all, patients. No patients exhibited prominent psychosis or dyskinesias, in keeping with other autoimmune encephalitides, such as NMDA‐receptor associated encephalitis, highlighting these features as a potential differentiator.

Symptom resolution occurred in all patients but was delayed where corticosteroids were not used. It thus appears that this clinical syndrome is highly steroid responsive, similar to other MOGAD phenotypes. Prompt recognition of the key clinical and radiographic features is therefore crucial to ensure prompt treatment and recovery [11]. Anecdotal evidence from our work suggests that delays to immunotherapy may result in slower symptomatic resolution.

Four patients in our case series had subsequent clinical relapse, suggesting that relapse risk is not materially different in patients with cerebral cortical encephalitis compared to other MOGAD syndromes. Two patients in our cohort relapsed while being MOG seropositive; however, two who relapsed were seronegative. Persistent anti‐MOG seropositivity is associated with increased risk of relapse in those with ADEM [6]. Further work is therefore needed to clarify whether MOG seropositivity is related to a risk of subsequent relapse in this patient group, and whether the overall risk of relapse reflects that of other clinical manifestations of MOGAD.

The higher prevalence of cerebral cortical encephalitis in patients of Asian ethnicity does not reflect background racial prevalence or the ethnoracial prevalence of patients with MOGAD in the United Kingdom. Cerebral cortical encephalitis has been commonly reported in Asian populations in the wider literature [9]. Any potential predilection for patients of this ethnicity warrants further investigation.

## CONCLUSIONS

This study describes the typical clinical features of cerebral cortical encephalitis. It also demonstrates key imaging and paraclinical investigation findings, with CSF analysis showing similar changes to those observed in other patients diagnosed with MOGAD. A combination of steroids and antiseizure medications led to relatively rapid symptomatic resolution in all patients except one, in whom steroids were withdrawn for clinical reasons. There should thus exist a low threshold for testing for MOG‐IgG in patients presenting with seizures and cortical changes on MRI. Longitudinal follow‐up of these patients is therefore needed to explore the risk of relapse and to determine whether this risk is affected by subsequent MOG antibody status.

## AUTHOR CONTRIBUTIONS

**S. Pace:** Investigation; writing – original draft; methodology; writing – review and editing; formal analysis; data curation. **S. Messina:** Investigation; writing – review and editing; data curation. **B. Chen:** Investigation; writing – review and editing. **R. Tanasescu:** Investigation; writing – review and editing. **A. Papathanasiou:** Investigation; writing – review and editing. **C. S. Constantinescu:** Investigation; writing – review and editing. **M. I. Leite:** Investigation; writing – review and editing; data curation. **M. D. Willis:** Investigation; writing – review and editing. **J. A. Johnston:** Investigation; writing – review and editing. **J. Palace:** Investigation; writing – review and editing; conceptualization; data curation. **R. Dobson:** Conceptualization; writing – review and editing; investigation; supervision; methodology.

## CONFLICT OF INTEREST STATEMENT

R.T. has received support from UK MRC (CARP MR/T024402/1) and has no other conflict of interest related to current work. R.D. has received honoraria for speaking and/or traveling from Biogen, Merck, Roche, Teva, Janssen, and Sanofi. She has served on advisory boards for Roche, Biogen, Janssen, Sandoz, and Merck. All honoraria for speaking, travelling, and advisory boards were paid into an institutional account and used to support research, open access publications, and training for group members. She has received grant support from Biogen, Merck, and Celgene. S.M. has received honoraria for speaking from UCB and travel grants from Merck, Roche, and Sanofi. J.P. has received support for scientific meetings and honoraria for advisory work from Merck Serono, Sandoz, Sanofi, Novartis, Chugai, Alexion, Clene, Roche, Medimmune, Amgen, Vitaccess, UCB, Mitsubishi, Amplo, and Janssen and grants from Alexion, Argenx, Roche, Medimmune, UCB, and Amplo Biotechnology. She holds patent ref. P37347WO, a licence agreement with Numares for multimarker MS diagnostics, and shares in AstraZeneca. Her group has been awarded an ECTRIMS fellowship and a Sumaira Foundation grant to start later this year. She worked as a Charcot fellow in Oxford during 2019–2021. She acknowledges partial funding to the trust by Highly Specialized Services NHS England. She is on the medical advisory boards of the Sumaira Foundation and MOG project charities, is a member of the Guthy Jackon Foundation Charity, is on the board of the European Charcot Foundation, is a member of the steering committee of MAGNIMS and the UK NHSE IVIG Committee, is chair of the NHSE neuroimmunology patient pathway, and has been an ECTRIMS council member on the educational committee since June 2023. She is a recent or current advisory member of the Association of British Neurology groups for multiple sclerosis, neuroinflammation, and neuromuscular diseases. J.A.J. has received honoraria for speaking and travelling from UCB and Eisai. M.I.L. is funded by the NHS (Myasthenia and Related Disorders Service and National Specialised Commissioning Group for Neuromyelitis Optica, UK) and by the University of Oxford, UK. She has been awarded research grants from UK associations for patients with myasthenia and with muscular disorders (Myaware and Muscular Dystrophy UK, respectively) and the University of Oxford. She has received speaker honoraria or travel grants from Biogen, Novartis, UCB Pharma, and the Guthy‐Jackson Charitable Foundation. She serves on scientific or educational advisory boards for argenx, UCB Pharma, and Viela Bio/Horizon Therapeutics/Amgen. S.P., M.D.W., A.P., B.C., and C.S.C. do not report any conflicts of interest related to the current work.

## Data Availability

The data that support the findings of this study are available on request and application via the Oxford NMO research database. The data are not publicly available due to privacy or ethical restrictions.
